# Anticipatory Grief and Associated Factors Among Family Caregivers of Patients with Palliative Needs in Portugal: A Community-Based Cross-Sectional Study

**DOI:** 10.3390/healthcare14203435

**Published:** 2026-10-14

**Authors:** Gisele Reami, Ana Ramos, Ana Querido, Maria dos Anjos Dixe, Carlos Laranjeira

**Affiliations:** 1 School of Health Sciences, University of Leiria and Oeste, Rua General Norton de Matos, 2411-901 Leiria, Portugal; gisele.reami@hotmail.com (G.R.); ana.claudiaeramoss@gmail.com (A.R.); ana.querido@ulo.pt (A.Q.); maria.dixe@ulo.pt (M.d.A.D.); 2 Centre for Innovative Care and Health Technology (ciTechCare), Campus 5, Rua das Olhalvas, 2414-016 Leiria, Portugal; 3 RISE Health Research Group, University of Porto, 4200-450 Porto, Portugal; 4 Comprehensive Health Research Centre (CHRC), University of Évora, 7000-801 Évora, Portugal

**Keywords:** family caregiver, anticipatory grief, caregiver burden, palliative care, community-based care, psychosocial factors

## Abstract

**Background/Objectives**: Anticipatory grief among family caregivers of individuals with palliative care needs is a complex and multidimensional phenomenon that remains underexplored in the Portuguese context, despite the central role of informal caregivers in care provision. Understanding the factors associated with this experience is essential for promoting caregivers’ well-being and the quality of palliative care. This study aims to assess anticipatory grief and associated factors among family caregivers of people with palliative needs at home in Portugal. **Methods**: An exploratory cross-sectional and descriptive-correlational study was conducted with a sample of 351 family caregivers in Portugal. Data were collected through an e-survey, including sociodemographic variables, the Marwit-Meuser Caregiver Grief Inventory—Short Form (MM-CGI-SF), the Zarit Burden Interview, the Family Needs Inventory (FIN), the Edmonton Symptom Assessment Scale (ESAS-r), and the Palliative Performance Scale (PPS). Data analysis was performed using descriptive statistics, correlation analysis, and multiple linear regression. The study followed the STROBE checklist. **Results**: Family caregivers presented a mean anticipatory grief score of 56.90 ± 13.84. Approximately 55.3% of the participants showed severe burden levels. Anticipatory grief was significantly associated with lower functional status of the care recipient, greater caregiver burden, higher intensity of emotional symptoms, and lower satisfaction of family needs. The multiple regression model explained 55.2% of the variance in anticipatory grief (R^2^ = 0.552). In the adjusted model, impact of caregiving, care-related expectations, functional status, fulfilment of family needs, caregiver age, perceived self-efficacy, and caregiver-proxy-rated depressive symptoms were significantly associated with anticipatory grief, with impact of caregiving and care-related expectations showing the largest standardized regression coefficients. **Conclusions**: Although exploratory in nature, the findings suggest that anticipatory grief represents an important aspect of the caregiving experience among family caregivers of individuals with palliative care needs. Early recognition of anticipatory grief, caregiver burden, and unmet support needs may facilitate the development of tailored interventions designed to alleviate psychological distress, enhance caregivers’ preparedness, and improve the quality of family-centered care.

## 1. Introduction

Currently, a large proportion of deaths worldwide is attributed to chronic or incurable diseases [1]. Of these, non-communicable diseases accounted for 43 million deaths per year, including 18 million premature deaths occurring before the age of 70 [2,3]. Given the projected population growth and increased longevity and ageing, the number of deaths and disability-adjusted life years resulting from all causes is expected to increase overall [4].

In this context, Palliative Care (PC) plays an increasingly important role in meeting the needs of people with life-threatening illnesses and their families. In Portugal, there has been significant progress in expanding the availability of specialized PC and, within the European context, the country has stood out for its continuous growth in the provision of these services [5]. The World Health Organization classifies Portugal as a country at an advanced stage of integration of PC into health systems [6].

However, despite these advances, there are still important gaps in access to PC and in structural and professional coverage, compromising the quality of life of patients and their families [7,8,9,10]. Recent estimates indicate that more than 70% of people with palliative needs still do not receive these services on time [11].

Among the challenges are fragmentation across different levels of care, inflexibility of care models, limited territorial adaptability, and weaknesses in professional training [9,10]. The persistence of misconceptions and limited understanding of PC, often associated solely with end-of-life care, represent an additional barrier to ensuring necessary care [12]. This is all the more significant given the regional inequalities in access to community PC teams. Consequently, many families assume a central role in managing and sustaining care at home, often without the technical and emotional support necessary to cope with the demands of caregiving [13].

Therefore, PC requires an integrated approach that includes not only the person with advanced illness but also their family members, who are recognized as central elements in the care process [6]. Studies show that family caregivers prefer two types of support: assistance focused on the care and well-being of the person at the end of life, and support directed at themselves to help sustain caregiving over time.

As the disease progresses, caregivers report a need for more intense and continuous support, reinforcing the importance of ongoing assessments and interventions tailored to individual preferences [14]. Well-prepared caregivers contribute to reduced use of healthcare services, such as hospital admissions and emergency department visits. To achieve this, healthcare professionals and services must provide structured support, technical knowledge, effective communication, and active inclusion in the therapeutic process, recognizing caregivers both as recipients and partners in care [15,16,17].

Caregivers commonly experience high levels of burden and emotional strain, often neglecting their own needs in favor of the well-being of the care recipient [18]. Recent studies indicate that family caregivers commonly experience moderate to severe levels of burden, although some caregivers show no signs of burden, highlighting the diversity of informal caregiving experiences [18,19].

Caregiver burden may be related to both objective factors—such as time constraints, physical effort, financial costs, and impacts on well-being—and subjective and emotional factors, as caregiving involves intense emotional and cognitive engagement that is often unrecognized [19].

Depression is more prevalent among caregivers (30%) than among patients with advanced illness (9%), which has contributed to the characterization of caregivers as “hidden patients” [20]. Furthermore, close contact with the care recipient and the frequent provision of care increase vulnerability to psychological distress, highlighting the need for targeted interventions that promote emotional support, grief management, and resilience-building [21].

The needs of family caregivers of individuals with chronic illness have been widely examined in the literature, with several reviews synthesizing existing evidence [22,23]. Early research in this field primarily addressed the needs of patients and their family caregivers simultaneously [24]. These studies identified several key domains of need, including information and communication, physical care, psychological support, social support, spiritual care, daily living assistance, and access to professional services. More recent reviews focusing specifically on caregivers of patients with particular diagnoses have further highlighted a broad range of needs, encompassing informational, physical, psychological, social, spiritual, financial, and care-related service requirements [23,25].

From the moment of diagnosis to the progressive worsening of the patient’s physical and cognitive limitations, caregivers experience successive and cumulative losses, including an increasing awareness of finitude. These experiences are often associated with significant psychological distress and require continuous adaptation [26,27]. This set of experiences can be understood in light of the concept of anticipatory grief, which describes the emotional and cognitive reactions that emerge before the actual loss, in contexts of progressive illness and imminent threat of death [28]. More than a static process, it is a dynamic experience that involves ongoing psychological reorganization in response to successive losses [29,30].

Although initially understood as a potentially protective mechanism, capable of mitigating the emotional impact associated with the death of individuals with life-threatening illnesses, more recent evidence indicates that this process may also be associated with greater psychological distress among caregivers. Thus, anticipatory grief may, on the one hand, constitute a source of prolonged burden and, on the other hand, represent an opportunity for learning, sharing, and strengthening emotional bonds, contributing to a gradual adaptation to loss and to the reduction in complications in bereavement after the loss [26,31,32]. Despite the growing recognition of the role of family caregivers in PC, studies that explore, in an integrated manner, the experience of anticipatory grief and associated factors remain limited. To address this knowledge gap, the present study aims to assess anticipatory grief and associated factors among family caregivers of people with palliative needs at home in Portugal.

## 2. Materials and Methods

### 2.1. Study Design

An exploratory cross-sectional, descriptive-correlational study was conducted. To ensure transparency and clarity in the reporting of methods and results, the STROBE (Strengthening the Reporting of Observational Studies in Epidemiology) checklist was followed [33].

### 2.2. Participants and Recruitment

Caregivers were recruited through an online survey distributed across different regions of Portugal. The e-survey was disseminated via social media platforms (Facebook and Instagram) and shared through digital networks of community settings, including churches, libraries, grocery stores, disease-specific associations, and healthcare institutions. A mixed sampling strategy was employed. Initially, convenience sampling was used, with the following inclusion criteria: (a) primary informal caregivers of individuals in a palliative situation (defined as a person who provides continuous, unpaid care to someone in a dependent condition, including spouses, partners, relatives up to the fourth degree, or individuals without kinship ties but with a proven relationship of mutual support and shared resources, regardless of whether they share the same household) [34]; (b) aged 18 years or older; (c) with adequate proficiency in Portuguese; and (d) having performed the caregiving role for at least three months. Exclusion criteria included non-primary caregivers and those with cognitive impairments that could compromise comprehension and responses to the assessment instruments.

For the purposes of this study, individuals with PC needs were defined as persons living with an advanced, progressive, or incurable illness associated with significant symptom burden, functional decline, or dependency requiring ongoing support and care. Eligibility was determined based on caregivers’ reports of the care recipient’s health condition and care needs. Clinical confirmation through medical records or enrolment in specialist palliative care services was not required.

Data collection was further complemented through snowball sampling, drawing on the personal networks of informal caregivers. Additionally, contacts were established with specialized community palliative care teams across the country, as well as with institutions and associations supporting individuals with chronic illness and their caregivers. Through these teams and services, potential participants were informed about the study and, if they agreed to participate, invited to contact the research team. This strategy aimed to facilitate the recruitment of participants with direct experience of the phenomenon under study and to increase the sample size.

To ensure adequate statistical power, an a priori power analysis was conducted using G*Power version 3.1.9.7 for a fixed-model multiple linear regression. Assuming 11 predictors, an α level of 0.05, statistical power of 0.80, and a conservative small-to-medium effect size (f^2^ = 0.10), the minimum required sample size was estimated at 305 participants. To account for potential invalid or incomplete responses, an additional 10% was added, resulting in a target sample size of at least 336 participants.

A total of 370 responses were received during the study period. Of these, 19 were excluded because of invalid submissions. The final analytical sample comprised 351 caregivers.

### 2.3. Data Collection

Data collection took place between May 2025 and September 2025. The e-survey required approximately 15 min to complete and consisted of six sections:

**I—Sociodemographic characterization of informal caregivers**—age [years], sex [female/male], marital status [married/common-law partnership; single; divorced/separated; widowed], educational level [no formal education; basic education; secondary education; higher education], and relationship to the care recipient [partner/spouse; son/daughter; mother/father; grandson/granddaughter; brother/sister; other].

**II—Assessment of the care recipient’s symptom intensity** using the Edmonton Symptom Assessment Scale (ESAS-r) [35], validated for European Portuguese by Bernardo [36]. The ESAS is a widely used symptom assessment tool in palliative care, applicable across different care settings, including hospitals, nursing homes, and home care, supporting a systematic and person-centered evaluation of symptom burden. The instrument comprises nine items assessing commonly reported symptoms in palliative care: pain, fatigue, nausea, depression, anxiety, drowsiness, appetite loss, well-being, and shortness of breath. Each symptom is rated on a numerical visual scale from 0 to 10, where 0 indicates absence of the symptom and 10 represents the worst possible intensity or distress. Because the survey was completed by family caregivers, all ESAS-r ratings reflected caregiver-proxy assessments of the care recipient’s symptoms. In the present study, the scale demonstrated a Cronbach’s alpha of 0.794.

**III—Assessment of care recipient’s functional decline** using the Palliative Performance Scale (PPS), developed by the Victoria Hospice Society [37] and validated for European Portuguese by Sereno [38]. This tool provides a rapid description of the patient’s current functional status. It assesses five domains: ambulation, activity level and evidence of disease, self-care, intake, and level of consciousness. The scale classifies patients according to their level of functionality, ranging from 0% (death) to 100% (full functionality). As data collection was conducted through an online survey, caregivers provided self-reported information for each of the five PPS domains. These responses were subsequently reviewed by the research team, and PPS scores were assigned according to the published scoring criteria [37,38].

**IV—Family Inventory of Needs (FIN)** [39,40], validated for Portugal by Areia, Major, and Relvas [41]. This questionnaire consists of 20 items assessing family needs. Each item is rated across two subscales: (a) Importance of Care Needs, which evaluates the perceived importance of each need using a Likert scale from 1 (“Not important”) to 5 (“Extremely important”); and (b) Fulfillment of Care Needs, which assesses the degree to which important needs are met, using a Likert scale from 1 (“Not satisfied”) to 3 (“Satisfied”). In the present study, the Importance of Care Needs and Fulfillment of Care Needs subscales showed Cronbach’s alpha values of 0.924 and 0.960, respectively.

**V—Zarit Burden Interview (ZBI)** [42], Portuguese version by Sequeira [43]. This instrument assesses both objective and subjective burden associated with informal caregiving, considering multiple dimensions such as physical and mental health impact, changes in social and personal life, financial effects, emotional well-being, and the quality of the relationship with the care recipient. The scale comprises 22 items rated on a 5-point Likert scale ranging from 1 (“Never”) to 5 (“Nearly always”), yielding a total score between 22 and 110, with higher scores indicating greater perceived burden. The items are organized into four factors: Impact of caregiving (items 1, 2, 3, 6, 9, 10, 11, 12, 13, 17 and 22); Interpersonal relationship (items 4, 5, 16, 18 and 19); Expectations regarding care (items 7, 8, 14 and 15); and Sense of self-efficacy (items 20 and 21). The applied cut-off points were those established in the Portuguese version: ≤45 = no burden; 46–56 = mild burden; ≥57 = high burden. In this study, the scale showed a Cronbach’s alpha of 0.895.

**VI—Marwit-Meuser Caregiver Grief Inventory**—Short Form (MM-CGI-SF) [44] using the Portuguese version validated by Areia, Major, and Relvas [45]. The MM-CGI-SF is an 18-item questionnaire rated on a 5-point Likert scale (1-strongly disagree to 5-strongly agree), designed to assess anticipatory grief experiences among adult family caregivers (>18 years) of individuals with advanced illness. Higher scores on the total grief scale represent greater experiences of grief [45]. Given that the factor structure of the Portuguese version of the MM-CGI-SF lacks factorial equivalence to the original instrument [45], the interpretation of subscale scores was considered potentially unreliable. Therefore, only the total score was retained for this study, providing a more robust measure of caregiver grief. In the present study, the global scale demonstrated Cronbach’s alpha of 0.894.

### 2.4. Data Analysis

Data analysis was performed using the SPSS 31.0 software package (SPSS Inc., Chicago, IL, USA). Percentage distributions and Cronbach’s alpha values for the scales and their sub-dimensions were calculated to evaluate the data. The Shapiro–Wilk test was employed to assess the normality of the scale scores. Despite minor departures from normality in some variables, the distributions were considered sufficiently symmetrical to justify the use of parametric statistical procedures, particularly given the large sample size. Pearson’s correlation coefficient was calculated to evaluate the linear relationship between two normally distributed continuous variables. Correlation coefficients were interpreted as follows: values between 0.00 and 0.25 indicated weak correlations; between 0.25 and 0.50, moderate correlations; between 0.50 and 0.75, strong correlations; and values above 0.75 indicated very strong correlations [46]. An independent samples *t*-test was used to compare mean values between two subgroups. For comparisons involving three or more subgroups, a one-way analysis of variance (ANOVA) was conducted, followed by Tukey’s post hoc test to identify specific group differences. The effect size was calculated using Hedges’ g and interpreted as a small effect if 0.2 < g < 0.5; a medium effect if 0.5 ≤ g ≤ 0.8; and a large effect if g > 0.8 [47]. Multiple linear regression analysis was conducted to evaluate the effects of family needs, caregivers’ burden and care recipient symptoms on the anticipatory grief scores of family caregivers. The factors that were significant in the univariate and correlation analyses were entered into the model as independent variables, with anticipatory grief as the dependent variable. Given the exploratory nature of the study, candidate variables for the multivariable model were identified based on their bivariate associations with anticipatory grief, while also considering their conceptual relevance to the study framework. These variables were entered simultaneously into a multiple linear regression model using the forced-entry method. The resulting coefficients therefore represent associations adjusted for the other variables included in the model. Multicollinearity diagnostics indicated no evidence of substantial collinearity among the independent variables, with variance inflation factor (VIF) values below 5 and tolerance values above 0.30 for all variables [46,48]. Furthermore, the Durbin–Watson test was utilized to examine the presence of autocorrelation in the residuals of the regression model. A statistical significance level of *p* < 0.05 was established.

### 2.5. Ethical Considerations

The study was conducted in accordance with international ethical standards for research involving human participants. Formal approval was obtained from the Ethics Committee of the University of Leiria and Oeste (CE/IPLeiria/11/2025 approved on 21 February 2025). All participants received written information detailing the study objectives, procedures, the voluntary nature of participation, and the measures taken to ensure the protection of personal data. Informed consent was obtained before participation, and participants were assured of their right to withdraw consent or discontinue participation at any time without any consequences. No financial incentives were provided for participation in the study.

## 3. Results

### 3.1. Sample Description

Participants had a mean age of 55.90 ± 13.37 years, ranging from 20 to 89 years (Table 1). Most caregivers were female (81.8%; n = 287). Regarding marital status, most participants (63.5%; n = 223) were married or living in a common-law partnership. In terms of education, 37.9% (n = 133) had completed basic education. Concerning living arrangements, 60.1% (n = 211) of caregivers cohabited with the care recipient.

With respect to the caregiving relationship, most caregivers were sons or daughters (37.0%; n = 130), followed by parents (26.2%; n = 92) and partners or spouses (19.9%; n = 70).

Functional status, assessed through the PPS, yielded a mean score of 51.23 ± 15.97, indicating a moderate level of overall functioning, with variability across participants. This suggests differing degrees of dependency among care recipients.

In terms of symptom burden, moderate to high mean scores were observed for fatigue (5.92 ± 2.87), pain (4.79 ± 3.18), and drowsiness (4.96 ± 2.96), as well as for psychological symptoms, including depression (5.45 ± 3.00) and anxiety (5.26 ± 3.02).

### 3.2. Anticipatory Grief, Family Needs and Caregiver’s Burden Among Participants

The results for the assessed variables, presented in Table 2, indicate moderate to substantial levels across several dimensions related to symptoms, family needs, anticipatory grief, and caregiver burden. Regarding anticipatory grief, the mean score was 56.90 ± 13.84. In terms of family needs, participants attributed notable levels of importance to their needs (93.55 ± 8.94), which contrasts with a substantially lower mean level of needs satisfaction (49.76 ± 9.71). This discrepancy suggests that needs are perceived as highly important but are not adequately met. The overall caregiver burden showed a mean score of 59.09 ± 14.92. When analyzed according to burden categories, more than half of the caregivers (n = 194; 55.3%) experienced severe burden, followed by mild burden (n = 92; 26.2%). Only 18.5% of participants (n = 65) reported no burden.

### 3.3. Correlation Analysis Between Study Variables

Based on Table 3, the findings indicate that higher levels of grief are associated with lower functional status of the care recipient, as demonstrated by the significant negative correlation with the PPS (global score). However, given the cross-sectional design, it is not possible to determine whether this association reflects disease progression, proximity to death, or other clinical circumstances. Grief also increases slightly with caregiver age, although this association was low. Anticipatory grief showed positive and significant associations with care recipient symptoms such as depression, anxiety, and overall distress, which indicates that higher levels of psychoemotional suffering are related to a greater intensity of anticipatory grief in caregivers.

Regarding needs, a strong negative association was found between fulfilment of care needs and grief, suggesting that caregivers who perceive their needs as less satisfied experience higher levels of grief. In contrast, the importance attributed to care needs did not show a significant association with grief.

One of the most relevant findings concerns caregiver burden, which showed strong positive correlations with grief, as well as with global burden and its specific dimensions. These findings highlight a close relationship between the experience of grief and the self-perception of burden.

### 3.4. Associations Between Anticipatory Grief and Sociodemographic and Caregiving Variables

Women presented a mean grief score of 57.03 (SD = 13.42), whereas men showed a slightly lower mean score of 56.38 (SD = 15.71). The independent samples Student’s *t*-test indicated that this difference was not statistically significant (t = 0.341; *p* = 0.734), demonstrating that there are no significant differences in grief levels between female and male caregivers.

The results indicate statistically significant differences in grief among different types of relationships with care recipients (F = 5.788; *p* < 0.001). Post hoc testing showed that caregivers who are partners/spouses exhibit the highest levels of grief. This group consistently stands out as having significantly higher grief levels compared to sons/daughters (mean difference = 6.71; *p* = 0.012), grandchildren (mean difference = 10.52; *p* = 0.013), and other relatives (mean difference = 14.05; *p* < 0.001). Effect size analysis using Hedges’ g revealed two distinct patterns of difference in anticipatory grief. The comparison between sons/daughters and grandchildren showed a moderate-magnitude difference (g = 0.494; 95% CI [0.200, 0.700]), with sons/daughters reporting noticeably higher levels of anticipatory grief. In contrast, the “other relatives” group differed from both of these groups to a much greater extent, showing a large-magnitude effect (g = 1.079; 95% CI [0.604, 1.549]). This wider confidence interval, however, suggests less precision in the estimate, likely reflecting the smaller sample size of that subgroup.

Although the overall analysis of variance indicated a significant association between residential distance and anticipatory grief, post hoc comparisons did not reveal statistically significant differences between individual groups. While a tendency toward higher grief scores was observed among caregivers living closer to the care recipient, this pattern should be interpreted with caution and regarded as exploratory given the lack of significant pairwise contrasts.

### 3.5. Factors Associated with Anticipatory Grief

A multiple linear regression analysis was conducted to examine the association between anticipatory grief and variables that showed statistically significant differences (Table 4). Eleven variables were simultaneously entered into the multiple linear regression model. After excluding cases with missing data, the final analysis comprised 337 participants. Collinearity diagnostics for the variables retained in the model showed VIF values ranging from 1.10 to 2.51 and tolerance values above 0.30. The Durbin–Watson statistic was 1.785, supporting the independence of residuals. The total ZBI score was excluded from the multivariable model because of perfect multicollinearity with the ZBI subscale scores (tolerance = 0.000). The ZBI subscale scores were retained to allow examination of the specific dimensions of caregiver burden associated with anticipatory grief. The overall regression model was statistically significant [F(11, 325) = 36.43, *p* < 0.001] and explaining a substantial proportion of the variance in anticipatory grief (R^2^ = 0.552; adjusted R^2^ = 0.537). Analysis of the regression coefficients revealed that several variables were significantly associated with anticipatory grief. Perceived impact of caregiving was the variable most strongly associated with anticipatory grief (β = 0.439, B = 0.634, *p* < 0.001), with higher perceived impact being associated with higher anticipatory grief scores. Expectations regarding care were also positively associated with anticipatory grief (β = 0.279, B = 1.257, *p* < 0.001). In contrast, functional status (PPS) was negatively associated with anticipatory grief (β = −0.159, B = −0.142, *p* < 0.001), with lower functional status being associated with higher grief scores. Similarly, greater fulfillment of care needs was associated with lower anticipatory grief (β = −0.092, B = −0.132, *p* = 0.028).

Perceived self-efficacy was positively associated with anticipatory grief, although with a smaller effect size (β = 0.102, B = 0.635, *p* = 0.015). Age (β = 0.095, B = 0.099, *p* = 0.016) and depressive symptoms (feeling sad) (β = 0.109, B = 0.509, *p* = 0.024) were also significant positive factors. Interpersonal relationship, anxiety (feeling nervous), sleepiness (*p* = 0.051), and general well-being (*p* = 0.054) did not reach statistical significance, although the latter two variables approached the significance threshold.

## 4. Discussion

Given the limited evidence examining anticipatory grief and its associated factors among family caregivers in home palliative care settings, this study aimed to assess anticipatory grief and identify factors associated with its occurrence among family caregivers in Portugal. The findings showed substantial variability in anticipatory grief scores, alongside considerable caregiver burden and a marked discrepancy between the importance attributed to caregivers’ needs and their perceived fulfillment. Together, these results highlight the multidimensional challenges faced by family caregivers and reinforce the need for comprehensive support strategies throughout the illness trajectory.

The co-occurrence of elevated anticipatory grief, substantial caregiver burden, and unmet support needs suggests that anticipatory grief is embedded within the broader caregiving experience and shaped by the cumulative demands associated with providing care. Consistent with the previous literature, anticipatory grief is not merely a cognitive anticipation of loss [49], but primarily a process of ongoing psychological distress that unfolds throughout the course of the illness [50]. Family caregivers often experience significant emotional impact related to the physical and psychological demands of caregiving, as well as uncertainty regarding the clinical trajectory of the disease [51]. Furthermore, anticipatory grief may be understood as a response to a series of ongoing losses that occur before death itself, including changes in family roles, reduced reciprocity in relationships, loss of future expectations, and witnessing the progressive decline of a loved one. These cumulative losses may be associated with feelings of sadness, helplessness, fear, and uncertainty, which are frequently reported alongside elevated emotional burden. At the same time, caregiving-related stress is associated with notable levels of anxiety and depression, which may coexist with anticipatory grief and other indicators of psychological distress during the illness trajectory [52]. These findings are particularly relevant, as caregivers’ psychological distress not only compromises their quality of life and caregiving capacity but is also associated with poorer patient outcomes, including early institutionalization, increased use of emergency services, and higher mortality [53,54].

Complementarily, the results support that greater levels of anticipatory grief were associated with lower functional status of the care recipient and greater intensity of psychological symptoms. These findings are consistent with the literature, indicating that progressive functional decline and proximity to death intensify caregivers’ emotional distress. At this stage, patients often experience significant physical and psychological symptoms, such as fatigue, pain, anxiety, and depression, requiring continuous assistance with daily activities [55,56,57]. As patients become increasingly dependent, caregivers must assume additional responsibilities, often resulting in physical exhaustion, reduced social participation, and disruption of personal and professional activities. These demands are often accompanied by reduced opportunities for self-care, restrictions on daily activities, and lower perceived well-being among caregivers [58,59]. Moreover, witnessing the progressive deterioration of a loved one’s physical and psychological condition may be associated with greater feelings of helplessness and awareness of the approaching loss, which may correspond to higher levels of anticipatory grief.

Our findings reinforce evidence that caregiver burden is closely related to physiological and psychosocial stress resulting from continuous care. Previous studies have also shown that both personal stress and caregiver role-related stress are positively associated with anticipatory grief [60,61,62]. The association observed in this study suggests that burden and anticipatory grief may not be independent phenomena, but rather interconnected experiences that mutually reinforce one another. Higher caregiving demands were associated with greater emotional exhaustion, while higher levels of anticipatory grief tended to coexist with lower perceived coping resources and resilience. Additionally, the literature indicates that caregivers with closer relational proximity—particularly spouses and primary caregivers—are more vulnerable to the development of anticipatory grief and psychological distress than other family caregivers, including symptoms of anxiety and depression, especially when associated with high levels of caregiving burden [52,63]. Similarly, the perception of important unmet needs by healthcare professionals and dysfunctional family functioning may contribute to psychological morbidity [52].

An important finding of the present study was the discrepancy between the importance attributed to caregivers’ needs and the low level of perceived satisfaction of those needs. This result suggests that, although caregivers recognize and value support across multiple domains, the assistance received may not adequately address their expectations or requirements. Caregivers’ unmet needs may encompass emotional support, information regarding disease progression, symptom management, future planning, financial concerns, and assistance with decision-making. Unmet needs have been associated with higher levels of distress, uncertainty, feelings of unpreparedness, and poorer psychological outcomes among caregivers, and may coexist with greater anticipatory grief and caregiver burden.

Prior research has emphasized the importance of support networks in caregiving and of practical preparation for death through active processes of planning, informed decision-making, provision of adequate information, and effective communication by healthcare professionals with family members. These strategies, in turn, are associated with lower levels of anxiety and depression among caregivers [64]. Nevertheless, some findings suggest that, although caregivers generally appreciated the information provided by care teams, it was, in certain situations, perceived as insufficient [65]. Given that unmet informational and emotional needs may exacerbate uncertainty and distress, healthcare professionals should adopt a proactive approach to caregiver support. Therefore, healthcare professionals must conduct early and continuous assessment of caregivers’ needs and psychological status, promoting interventions that facilitate adaptation to the illness trajectory and reduce the impact of caregiver burden and anticipatory grief [60].

Taken together, these findings suggest the importance of integrating caregiver assessment into routine palliative care practice. Early identification of caregivers experiencing marked levels of burden, unmet needs, and anticipatory grief may help target supportive interventions focused on coping, emotional adjustment, and preparedness for illness progression and end-of-life transitions. Such family-centered approaches may not only improve caregivers’ well-being but also contribute to better outcomes for patients receiving palliative care at home [53,54].

### 4.1. Study Limitations

This study has several limitations that should be considered when interpreting the findings. First, the cross-sectional design limits the ability to establish causal or temporal relationships between anticipatory grief, caregiver burden, family needs, and clinical variables. Although lower patient functionality was associated with higher levels of anticipatory grief, the study did not collect longitudinal information regarding patients’ functional trajectories or disease progression. Consequently, it is not possible to determine whether reduced functionality reflected progression toward the end of life, a stable clinical condition, or other circumstances. Longitudinal studies are needed to better understand how changes in patients’ health status influence caregivers’ anticipatory grief over time.

Second, data were collected through self-reported measures, which may be subject to recall bias, response bias, and social desirability effects, potentially influencing the accuracy of the reported experiences. Moreover, the identification of PC needs was based on caregiver report and was not independently confirmed through clinical records or healthcare professionals, which may have resulted in heterogeneity in the study population.

Third, the use of an online questionnaire and a non-probabilistic sampling strategy may have introduced selection bias. Participation was restricted to caregivers with access to digital technologies and sufficient digital literacy, which may have led to the underrepresentation of older caregivers, individuals with lower educational attainment or socioeconomic status, and those experiencing greater caregiving demands. Consequently, the sample may overrepresent caregivers who are relatively more engaged, educated, technologically connected, or supported. These factors should be considered when interpreting the observed statistical associations, and the findings should be regarded as exploratory rather than representative of all family caregivers of individuals with palliative care needs in Portugal. Additionally, although the sample size was robust, the study was conducted within the Portuguese context and employed a non-probability sampling approach, limiting the generalizability of the findings to other populations, cultures, and healthcare systems.

Finally, other potentially relevant variables, such as caregivers’ prior mental health status, coping strategies, resilience, and availability of formal and informal social support, were not assessed and may also influence anticipatory grief. Future research should employ longitudinal designs and more representative sampling strategies to confirm these preliminary findings and to better understand the temporal dynamics of anticipatory grief. The inclusion of more diverse populations, both within and across countries, would strengthen the external validity of findings. Furthermore, incorporating additional psychosocial variables and qualitative data could provide deeper insight into the mechanisms underlying anticipatory grief and inform the development of tailored interventions for family caregivers.

### 4.2. Implications for Practice

These findings highlight the need for routine, early assessment of anticipatory grief, caregiver burden, and family needs within home palliative care, integrating validated tools into clinical practice. To mitigate anticipatory grief among family caregivers, evidence supports the implementation of early, multicomponent, and caregiver-centered interventions within palliative care. These should include routine screening for grief and burden, combined with psychoeducational support to normalize grief responses and provide clear information about disease progression and care expectations. Psychological interventions—such as counselling, support groups, and mindfulness-based strategies—can help reduce emotional distress and enhance coping [66]. Additionally, structured communication and advanced care planning, facilitated by healthcare professionals, may reduce uncertainty and improve preparedness [66,67]. Practical support measures, including respite care and skills training, are also essential to alleviate caregiver burden. Collectively, these approaches—delivered through an interdisciplinary framework—can promote emotional adaptation, reduce psychological morbidity, and improve both caregiver well-being and overall quality of care. In parallel, education and training programs should equip healthcare professionals with competencies in assessing and managing anticipatory grief and delivering family-centered care. At a policy level, the results support the integration of caregiver assessment into palliative care standards, greater recognition of caregivers as part of the unit of care, and the expansion of support services and resources for informal caregivers in the community. Future research should further evaluate the effectiveness of targeted interventions and examine contextual and cultural factors influencing caregivers’ experiences, particularly within underrepresented populations and different care settings.

## 5. Conclusions

This exploratory study characterized anticipatory grief and its associated factors among family caregivers of individuals with perceived palliative care needs. Overall, the findings suggest that anticipatory grief in family caregivers should not be understood solely as a response to the proximity of death, but rather as a multifactorial process shaped by clinical, emotional, and relational factors. Early identification of caregivers at greater psychological risk may facilitate the implementation of targeted interventions aimed at providing emotional support, strengthening coping strategies, improving communication with healthcare teams, and promoting adaptation to the processes of illness and impending loss. Furthermore, this study contributes to advancing scientific knowledge on the experiences of family caregivers within the Portuguese palliative care context. Recognizing anticipatory grief as a central dimension of care may not only enhance caregivers’ well-being and quality of life but also improve the quality and continuity of care delivered to individuals with palliative care needs.

## Figures and Tables

**Table 1:** Sociodemographic and caregiving details (N = 351).

Variables	Categories	N	%
Sex	Female	287	81.8
	Male	64	18.2
Marital Status	Married/common-law marriage	223	63.5
	Single	64	18.2
	Divorced/Separated	37	10.5
	Widowed	27	7.7
Academic qualifications	No formal education	13	3.7
	Basic education	133	37.9
	Secondary education	84	23.9
	Higher education	121	34.5
Distance between the caregiver’s residence and the family member	Lives in the same household	211	60.1
	Less than 20 min from the residence	103	29.3
	20 min or more from the residence	37	10.5
Relationship with the care recipient	Partner/Spouse	70	19.9
	Son/Daughter	130	37.0
	Mother/Father	92	26.2
	Grandson/Granddaughter	25	7.1
	Brother/Sister	8	2.3
	Other	26	7.4
Variables (Care recipients)	Categories	Mean	SD
Palliative Performance Scale (PPS)	Global score	51.23	15.97
Edmonton Symptom Assessment System (ESAS-r)	Pain	4.79	3.18
	Fatigue	5.92	2.87
	Drowsiness	4.96	2.96
	Nausea	1.97	2.81
	Appetite loss	4.06	3.09
	Shortness of breath/Dyspnea	2.37	2.97
	Depression (feeling sad)	5.45	3.00
	Anxiety (feeling nervous)	5.26	3.02
	Well-being (overall sense of well-being/ill-being)	5.18	2.59
	Another symptom	4.17	3.75

**Table 2:** Anticipatory grief, burden and caring needs of study participants (N = 351).

Variables	Median	Mean	SD
Anticipatory Grief (Total)	57	56.90	13.84
FIN—Importance of Care Needs subscale	97	93.55	8.94
FIN—Fulfillment of Care Needs subscale	53	49.76	9.71
Zarit Burden Interview (Total)	58	59.09	14.92
Impact of caregiving	29	29.02	9.58
Interpersonal relationship	10	10.21	3.79
Expectations regarding the care	15	14.88	3.14
Sense of self-efficacy	5	4.86	2.23

FIN—Family Inventory Needs; SD—Standard Deviation.

**Table 3:** Intercorrelations between study variables.

Variables		r	*p*
Age		0.124 *	0.021
PPS	Total score	−0.216 **	<0.001
ESAS-r	Pain	0.036	0.502
	Fatigue	0.063	0.242
	Drowsiness	0.126 *	0.018
	Nausea	0.097	0.070
	Appetite loss	0.095	0.076
	Shortness of breath/Dyspnea	0.081	0.129
	Depression (feeling sad)	0.175 **	0.001
	Anxiety (feeling nervous)	0.164 **	0.002
	Well-being (feeling of well-being)	0.200 **	<0.001
	Another symptom	0.056	0.383
FIN	Importance of Care Needs subscale	0.089	0.099
	Fulfillment of Care Needs subscale	−0.291 **	<0.001
ZBI	Impact of caregiving	0.642 **	<0.001
	Interpersonal relationship	0.449 **	<0.001
	Expectations regarding the care	0.573 **	<0.001
	Sense of self-efficacy	0.181 **	0.001
	Total caregiver burden	0.670 **	<0.001

FIN—Family Inventory Needs; ZBI—Zarit Burden Interview; ESAS-r—Edmonton Symptom Assessment Scale-revised; PPS—Palliative Performance Scale; * Correlation is significant at the 0.05 level (2-tailed); ** Correlation is significant at the 0.01 level (2-tailed).

**Table 4:** The effect of the independent variables on anticipatory grief.

Dependent Variable	Independent Variables		Model					95% CI		Tolerance	VIF	F	Model (*p*)	Adjusted R^2^	DW
			B	SE	β	*t*	*p*	Lower	Upper						
Anticipatory Grief	constant		23.201	6.219	-	3.731	**<0.001**	10.966	35.436	-	-	F(11.325) = 36.43	**<0.001**	0.537	1.785
	Age		0.099	0.041	0.095	2.429	**0.016**	0.019	0.180	0.907	1.103				
	FIN	Fulfillment of Care Needs subscale	−0.132	0.060	−0.092	−2.211	**0.028**	−0.250	−0.015	0.791	1.264				
	ZBI	Impact of caregiving	0.634	0.085	0.439	7.459	**<0.001**	0.467	0.801	0.398	2.514				
		Interpersonal relationship	−0.072	0.201	−0.020	−0.359	0.720	−0.468	0.324	0.453	2.206				
		Expectations regarding the care	1.257	0.203	0.279	6.201	**<0.001**	0.858	1.656	0.682	1.467				
		Sense of self-efficacy	0.635	0.261	0.102	2.436	**0.015**	0.122	1.148	0.786	1.272				
	ESAS-r	Drowsiness	−0.388	0.198	−0.083	−1.960	0.051	−0.778	−0.002	0.772	1.295				
		Depression (feeling sad)	0.509	0.224	0.109	2.273	**0.024**	0.068	0.950	0.594	1.682				
		Anxiety (feeling nervous)	−0.129	0.217	−0.028	−0.593	0.554	−0.556	0.298	0.615	1.626				
		Well-being (feeling of well-being)	0.459	0.237	0.085	1.935	0.054	−0.008	0.925	0.712	1.405				
	PPS	Total of PPS	−0.142	0.038	−0.159	−3.779	**<0.001**	−0.217	−0.068	0.782	1.279				

FIN—Family Inventory Needs; ZBI—Zarit Burden Interview; ESAS-r; Edmonton Symptom Assessment Scale-revised; PPS—Palliative Performance Scale; B—unstandardized regression coefficient; SE—standard error; β—standardized regression coefficient; DW—Durbin–Watson; CI—confidence interval; VIF—variance inflation factor; F = Fishers F test; R^2^ = adjusted coefficient of determination; Bold indicates that *p* < 0.05.

## Data Availability

The dataset contains sensitive information collected from family caregivers regarding anticipatory grief, emotional experiences, and related psychosocial variables. Although all data were anonymized, ethical approval and informed consent procedures did not include unrestricted public data sharing. Therefore, access to the dataset is restricted to protect participant confidentiality and privacy and to comply with ethical and data protection requirements. The data are available from the corresponding author upon reasonable request and subject to approval by the research team and compliance with the ethical conditions under which the study was conducted.

## Abbreviations

The following abbreviations are used in this manuscript:

ESAS-r	Edmonton Symptom Assessment System-revised
FIN	Family Inventory Needs
MM-CGI-SF	Marwit-Meuser Caregiver Grief Inventory—Short Form
PC	Palliative Care
ZBI	Zarit Burden Interview
